# Systemic juvenile idiopathic arthritis: The Great Ormond Street Hospital experience (2005–2021)

**DOI:** 10.3389/fped.2023.1218312

**Published:** 2023-09-12

**Authors:** C. M. Foley, D. McKenna, K. Gallagher, K. McLellan, H. Alkhdher, S. Lacassagne, E. Moraitis, C. Papadopoulou, C. Pilkington, M. Al Obaidi, D. Eleftheriou, P. Brogan

**Affiliations:** ^1^ Department of Paediatric Rheumatology, Great Ormond Street Hospital for Children NHS Foundation Trust, London, United Kingdom; ^2^ Department of Paediatric Rheumatology, University College London Great Ormond Street Institute of Child Health and Great Ormond Street Hospital for Children NHS Foundation Trust, London, United Kingdom

**Keywords:** Still’s disease, systemic JIA, biologic, IL-1 blockade, IL-6 blocking

## Abstract

Systemic juvenile idiopathic arthritis (sJIA) is a complex, systemic inflammatory disorder driven by both innate and adaptive immunity. Improved understanding of sJIA pathophysiology has led to recent therapeutic advances including a growing evidence base for the earlier use of IL-1 or IL-6 blockade as first-line treatment. We conducted a retrospective case notes review of patients diagnosed with sJIA over a 16-year period (October 2005–October 2021) at Great Ormond Street Hospital for Children. We describe the clinical presentation, therapeutic interventions, complications, and remission rates at different timepoints over the disease course. We examined our data, which spanned a period of changing therapeutic landscape, to try and identify potential therapeutic signals in patients who received biologic treatment early in the disease course compared to those who did not. A total of 76-children (female *n* = 40, 53%) were diagnosed with sJIA, median age 4.5 years (range 0.6–14.1); 36% (27/76) presented with suspected or confirmed macrophage activation syndrome. A biologic disease-modifying anti-rheumatic drug (bDMARD) alone was commenced as first-line treatment in 28% (*n* = 21/76) of the cohort; however, at last review, 84% (*n* = 64/76) had received treatment with a bDMARD. Clinically inactive disease (CID) was achieved by 88% (*n* = 67/76) of the cohort at last review; however, only 32% (24/76) achieved treatment-free CID. At 1-year follow-up, CID was achieved in a significantly greater proportion of children who received treatment with a bDMARD within 3 months of diagnosis compared to those who did not (90% vs. 53%, *p* = 0.002). Based on an ever-increasing evidence base for the earlier use of bDMARD in sJIA and our experience of the largest UK single-centre case series described to date, we now propose a new therapeutic pathway for children diagnosed with sJIA in the UK based on early use of bDMARDs. Reappraisal of the current National Health Service commissioning pathway for sJIA is now urgently required.

## Introduction

Systemic juvenile idiopathic arthritis (sJIA) is a complex, systemic inflammatory disorder. Historically known as Still's disease in recognition of its early description by George Frederic Still in 1897 (1), it is classified alongside other forms of juvenile idiopathic arthritis (JIA) using the International League of Associations for Rheumatology (ILAR) criteria (2). Arthritis is not always a presenting feature of sJIA, leading some to suggest we return to the nomenclature Still's disease. This would re-align with the adult form of the disease, adult onset Still's disease (AOSD), which differs only by age at first presentation (3), and would potentially reduce the risk of misdiagnosis of patients who first present with systemic features of the disease in the absence of arthritis.

sJIA can manifest varying degrees of arthritis but is distinct from other forms of JIA because of its prominent systemic inflammatory features. As such, there has been a move to consider sJIA as an autoinflammatory disease driven predominantly by dysregulation of the innate immune system. However, autoimmunity almost certainly still has a role to play in the pathogenesis since genome-wide association studies (GWAS) have established an unequivocal link between sJIA and the class II HLA region, in particular, HLA-DRB1*11 (4, 5)*.* Nigrovic attempted to reconcile these seemingly disparate views by proposing a biphasic hypothesis with an early autoinflammatory phase driven predominantly by interleukin 1 (IL-1), which if left unchecked drives a state of chronic adaptive immune activation involving effector T-cell activation (including resident synovial memory T cells), regulatory T-cell resistance, and Th-17 differentiation (4, 6).

Such a biphasic model is attractive and certainly would explain: (1) the observation that many children with sJIA begin with severe systemic features that can then evolve over months or years to a more chronic arthritis phenotype (6, 7); (2) the effectiveness of IL-1 blockade in some patients, particularly early in the disease (8); and (3) the propensity to macrophage activation syndrome (MAS), driven initially by IL-1 and IL-18, which in turn activate interferon-gamma (INF-γ) and IL-2 production from activated CD8 lymphocytes (9). Intriguingly, this biphasic model of disease also suggests that early treatment during the autoinflammatory phase might halt the progression to chronic adaptive immune activation, the so-called “window of opportunity” to treat sJIA (6). This model does not, however, currently explain why some patients respond better to IL-6 rather than IL-1 blockade (and vice versa) (6) nor does it yet suggest a viable biomarker to facilitate precision medicine (i.e., choice of IL-1 or IL-6 blockade) for individual patients with sJIA.

Aside from these unresolved questions, successful clinical trials of both IL-1 and IL-6 blockade leading to their regulatory approval for treatment of sJIA have undoubtedly transformed clinical outcomes by ameliorating systemic inflammation, preventing destructive arthritis, and reducing glucocorticoid toxicity (8, 10). Significant therapeutic efficacy and safety of tocilizumab (anti-IL-6 receptor antibody), anakinra (IL-1 receptor antagonist), and canakinumab (anti-IL-1beta antibody) are clearly demonstrated (10–12), and have also led to new insights into disease pathogenesis driven by IL-1 and IL-6 in sJIA (13, 14).

Despite these advances, not all countries have readily adopted these treatments into routine clinical care, largely due to the cost of these drugs. In England, current National Health Service (NHS) guidelines for the treatment of sJIA still mandate the use of the synthetic disease-modifying anti-rheumatic drug (sDMARD) methotrexate for at least 3 months before a biologic (anakinra or tocilizumab) may be considered (15–17). This is despite clear evidence from a randomised, placebo-controlled cross-over trial of methotrexate in children with sJIA demonstrating no significant reduction in systemic features or number of active joints with methotrexate, compared to placebo (18). The only exception is for patients in England that develop MAS, who may access anakinra without delay (15). The situation is even more challenging for AOSD, since adults are only granted access when they fail to respond to two sDMARDs (16), despite Nordström et al. demonstrating the superior beneficial effect of IL-1 blockade compared to sDMARD in an open, randomised, multi-centre study (19), and proven benefits of IL-6 blockade for both joint disease and systemic features in AOSD (20, 21). Finally, at the time of writing, canakinumab is not routinely commissioned in England for either sJIA or AOSD despite sufficient published evidence to support the use of this agent (22).

With this background in mind, we set out to describe our experience of sJIA over a 16-year period at Great Ormond Street Hospital for Children NHS Foundation Trust, London (GOSH), a tertiary referral paediatric rheumatology centre. Since this time period spanned a changing therapeutic landscape in the UK, we hypothesised that there could be therapeutic heterogeneity around the use of biologics for sJIA that might retrospectively reveal potential therapeutic signals in patients who received biologic treatment early in the disease course compared to those who did not. We therefore conducted a retrospective case notes review of children diagnosed with sJIA between October 2005 and October 2021 (inclusive) with specific emphasis on demography, clinical presentation, therapeutic interventions, complications, and remission rates at different timepoints over the course of the disease.

## Methods

### Electronic patient record search and patient inclusion

We used electronic institutional clinical record coding to identify all patients with a diagnosis of sJIA seen at GOSH over a 16-year period. Using the search terms sJIA, Still's disease, systemic onset JCR (juvenile chronic arthritis), and AOSD, we identified patients diagnosed with sJIA between 2 October 2005 and 21 October 2021 (inclusive). Medical notes were reviewed retrospectively. All patients with a final diagnosis of sJIA made by a consultant paediatric rheumatologist were included. Patients were excluded where the final diagnosis was malignancy or other sJIA mimic. This retrospective case notes review received full institutional approval (R&D: 21IH01), and as all data collected were anonymised, individual patient consent was not required.

### Data collection

Data recorded were anonymised and stored using an NHS computer on a password protected Microsoft Excel spreadsheet (version 16.16.27). Patient demographics including age at diagnosis and sex were recorded. Symptoms and signs present at diagnosis informing the ILAR sJIA classification criteria were documented (2): arthritis, fever, evanescent rash, lymphadenopathy, hepatomegaly, splenomegaly, and evidence of serositis. Data on the active joint count (AJC), presence of early morning stiffness (EMS), presence of uveitis as defined by the standardisation of uveitis nomenclature (SUN) working group (23), physician's global assessment (PGA) of overall disease activity on a visual analogue scale of 0–100 mm (0 = no activity; 100 mm = maximum activity), erythrocyte sedimentation rate (ESR, mm/h) and C-reactive protein (CRP, mg/L) were also captured. To obtain a retrospective quantitative measurement of the activity of systemic disease, components required to complete the systemic manifestation score (SMS) were recorded (24). The SMS ranges from 0 to 10, where 0 = absence of systemic manifestations and 10 = maximum activity of systemic manifestations. Points are assigned as follows: fever = 1 point if 37°C–38°C, 2 points if 38°C–39°C, 3 points if 39°C–40°C, 4 points if >40°C; rash = 1 point; generalised lymphadenopathy = 1 point; hepatomegaly and/or splenomegaly = 1 point; serositis = 1 point; anaemia (defined as haemoglobin  < 9 g/dl) = 1 point; platelet count >600 × 10^9^/L or ferritin >500 ng/ml = 1 point (Supplementary Figure S1). In patients where fever was documented in the notes, but a value not available, a score of 2 was awarded. A fever score of 1 was applied to cases where the child was apyrexial, but again no value available. Timepoints for data collection were at diagnosis, 3 months post diagnosis, 1 year post diagnosis, and last review. Clinical and laboratory data as outlined above were recorded for each timepoint. Information on the treatment initiated at diagnosis and ongoing or commenced at follow-up reviews was also recorded.

### Clinically inactive disease and remission definition

Clinically inactive disease (CID) was defined using the modified Wallace Criteria (25). CID required the absence of arthritis, EMS, systemic features, and uveitis; a PGA indicating no disease activity with a score of zero; and normalisation of ESR and CRP. The percentage of patients with CID at each timepoint was calculated. This was further subdivided to report CID depending on treatment status, i.e., (1) irrespective of any current treatment; (2) glucocorticoid-free (irrespective of other treatments received); and (3) remission, defined as clinically inactive disease, off all medication. These disease outcomes were specifically analysed with respect to receipt of IL-1 or IL-6 blockade^,^ within 3 months of diagnosis or not to investigate whether early commencement of biologic might influence the longer-term disease course. This 3-month time point was chosen pragmatically for three main reasons: (1) 3 months is a review point that we generally use in our routine clinical practice before deciding whether a treatment has been effective or not, and is thus amenable to data capture retrospectively in an outpatient review setting; (2) it is a realistic proxy of “early” when considering a therapeutic window of opportunity; and (3) 3-month review allowed us to compare and contrast with other published studies exploring early biologic use in sJIA (6, 8, 26).

### Adverse events of special interest

Complications of particular interest were captured. These were frequency of MAS, sJIA-associated lung disease (sJIA-LD), requirement for haematopoietic stem cell transplantation (HSCT), and deaths. Confirmed MAS was defined as MAS fulfilling the 2016 classification criteria (27); suspected MAS was assigned to those patients where this diagnosis was documented as being made in the electronic record, but without full documentary evidence satisfying these classification criteria. A diagnosis of sJIA-LD was documented when suggested by findings on high-resolution computed tomography (HRCT) in the absence of any alternative cause for these findings (28).

### Data handling and statistics

Descriptive statistics were reported as median and range or interquartile range (IQR) for continuous variables and as absolute frequencies and percentages for categorical variables unless otherwise specified. Comparisons of quantitative variables between two groups were made by the Mann–Whitney *U*-test. Categorical data were compared using Fisher's exact test. All statistical tests were two-tailed; *p* values <0.05 were considered significant. Data were analysed using Microsoft Excel (version 16.16.27) and the online resource Social Science Statistics (29).

## Results

### Demographics and clinical features at presentation

A total of 95 children were identified from the initial database search. On review of the records, 19 children were excluded. Sixteen of the 19 children had an alternate final diagnosis, e.g., other JIA subtype or vasculitis. Three children were not included as they were seen at GOSH for a second opinion but treated elsewhere.

The final cohort for analysis consisted of 76 patients (female *n* = 40, 53%), median age 4.5 years (range 0.6–14.1) at disease diagnosis. Table 1 summarises the clinical characteristics of the patients with sJIA. All patients (100%, *n* = 76) presented with fever. Fifty-nine (78%) patients fully met the ILAR classification criteria for sJIA at diagnosis. Of the remaining 17 not fulfilling the classification criteria, arthritis was absent at diagnosis in 15; the remaining two had fever and arthritis but did not have additional features to fully satisfy the criteria (2). Median time of follow-up at last review for the cohort was 4.7 years (range 0.2–16.0).

**Table 1:** Baseline characteristics of patients with sJIA and treatment overview.

Clinical information	Patients with sJIA (*n* = 76)
Demographics	
Age at diagnosis, years (range)	4.5 (0.6–14.1)
Sex, % female (*n* = male, female)	53% (36, 40)
Clinical manifestations	
Fever, *n* (%)	76 (100%)
Rash, *n* (%)	69 (91%)
Lymphadenopathy, *n* (%)	37 (49%)
Hepatomegaly, *n* (%)	11 (14%)
Splenomegaly, *n* (%)	17 (22%)
Serositis, *n* (%)	11 (14%)
Arthritis, *n* (%)	61 (80%)
Active joint count, *n* = 70 (range)	4 (0–26)
MAS^a^, *n* (%)	27 (36%) (confirmed, *n* = 20; suspected, *n* = 7)
Laboratory investigations	
ESR (mm/h), *n* = 48 (range)	120 (10–170)
CRP (mg/L), *n* = 54 (range)	95 (5–400)
Ferritin >500 μg/L (*n* = 59)	37 (63%)
Haemoglobin <9 g/dl (*n* = 59)	14 (24%)
Platelets >600 × 10^9^/L (*n* = 61)	18 (30%)
sJIA classification criteria and systemic manifestation score	
Met the classification criteria for sJIA	59 (78%)
Systemic manifestation score^b^ (*n* = 66)	4 (0–9)
Treatment received within 3 months of diagnosis	
Oral/IV glucocorticoids (%)	70 (92%)
Methotrexate (%)	36 (47%)
IL-1 blockade (anakinra)^c^, *n* (%)	21 (28%)
IL-6 blockade (tocilizumab)^c^, *n* (%)	6 (8%)
Other immune modulation^d^, *n* (%)	22 (29%)
Treatment at last review	
Time since diagnosis, years (range)	4.7 (0.2–16)
Oral/IV glucocorticoids (%)	6 (8%)
Methotrexate as sole DMARD (%)	8 (11%)
Current anakinra or tocilizumab^c^, *n* (%)	38 (50%; *n* = 20 anakinra; *n* = 18 tocilizumab)
Other treatment^e^, *n* (%)	14 (18%)
Biologic ever^f^, *n* (%)	64 (84%)

Median values presented along with range. Absolute frequencies presented along with percentages.

^a^ MAS at presentation. This has been subdivided to report number of children that presented with confirmed MAS, i.e., fulfilled the 2016 MAS classification criteria (25); and suspected MAS, i.e., where a diagnosis of MAS was documented in the electronic record, but without full documentary evidence satisfying the classification criteria.

^b^ SMS quantifies burden of systemic symptoms on a 0–10 scale. Includes five clinical elements (fever, evanescent rash, generalised lymphadenopathy, hepatomegaly and/or splenomegaly, and serositis) and two laboratory elements (anaemia, defined as Hb < 9 g/dl, and either platelet count >600 × 10^9^/L or ferritin >500 ng/ml). Each item, if present scores 1, apart from fever which is further characterised as follows: temperature 37°C–38°C (scores 1), 38°C–39°C (scores 2), 39°C–40°C (scores 3), and >40°C (scores 4) (23).

^c^ Irrespective of concomitant sDMARD or immunosuppressant.

^d^ Treated with an alternative agent(s): cyclosporine *n* = 12, intravenous immunoglobulin *n* = 6, emapalumab *n* = 2, etoposide *n* = 1, and etanercept *n* = 1.

^e^ Treated with an alternative agent(s): cyclosporine *n* = 3, adalimumab *n* = 3, HSCT *n* = 4, canakinumab *n* = 2, etanercept *n* = 1, and colchicine *n* = 1.

^f^ Received treatment with a biologic DMARD at some point throughout their disease course: IL-1 blockade, *n* = 35; IL-6 blockade, *n* = 26; IL-1 and IL-6 (sequentially), *n* = 3; anti-TNF, *n* = 8). IV, intravenous; IL, interleukin.

### Treatment and response

Table 1 summarises the treatments received. The majority (92%; 70/76) received glucocorticoids as part of initial drug management, either oral and/or intravenous. Methotrexate was commenced at diagnosis in 47% of cases (*n* = 36/76). Of these, 6/36 (8% of the total cohort) also received biologic therapy at diagnosis (methotrexate and anti-IL-1, *n* = 3; methotrexate and anti-IL-6, *n* = 3). Anti-IL-1 or anti-IL-6 without methotrexate was used as first-line DMARD within the first 3 months of diagnosis in 28% of the cohort (anti-IL-1, *n* = 18, anti-IL-6, *n* = 3). In contrast, at last review, 84% (*n* = 64/76) of the cohort had received treatment with at least one biologic DMARD (bDMARD) at some stage over their disease course [IL-1 blockade, *n* = 35; IL-6 blockade, *n* = 26; IL-1 and IL-6 (sequentially), *n* = 3; anti-TNF, *n* = 8].

Response to treatment is summarised in Table 2. CID was achieved by 88% (*n* = 67/76) of the cohort at last review, irrespective of current treatment status; 84% (*n* = 64/76) of children with CID were glucocorticoid-free at last review; and 32% (*n* = 24/76) were completely treatment-free (in remission) at last review. CID on treatment was achieved by 57% (43/76) of the cohort. Of these patients, 43% (*n* = 33/76) remained on either IL-1 (*n* = 19) or IL-6 (*n* = 14) blockade; nine patients remained on methotrexate, 7% (*n* = 5/76) of whom were on methotrexate alone; and 7% (5/76) achieved remission by alternative medications: four patients on anti-TNF therapy and one patient on cyclosporine alone.

**Table 2:** Clinically inactive disease following treatment for the whole sJIA cohort (*n* = 76).

	3 months *n* = 76	12 months *n* = 63	Last follow-up *n* = 76
	3.1 months (2.7–3.8)	12 months (11.1–13.3)	4.7 years (2.2–9.2)
Clinically inactive disease^a^ (%)	44/76 (58%)	44/63 (70%)	67/76 (88%)
Treatment-free clinically inactive disease (remission; %)	3/76 (4%)	3/63 (5%)	24/76 (32%)
Glucocorticoid-free clinically inactive disease^b^ (%)	14/76 (18%)	36/63 (57%)	64/76 (84%)

Median age presented along with interquartile range (IQR). Absolute frequencies presented along with percentages.

^a^ Clinically inactive disease as per the modified Wallace criteria (25) irrespective of current treatment status.

^b^ Clinically inactive disease as per the modified Wallace criteria (25), off glucocorticoids (irrespective of other treatments received).

At last review, 12% of the cohort (*n* = 9/76) had not attained CID. Chronic arthritis was observed in four patients (5%), one with an associated systemic rash. Four patients (5%) had ongoing fevers, two associated with evanescent rash. One child had the evanescent rash only.

### Timing of biologic and effect on rates of CID

To explore the hypothesis that there may be an early window of opportunity to treat in order to achieve long-standing CID, we looked at the timing of initiation of treatment with bDMARD from diagnosis and rates of CID at follow-up (Table 3). Of note, those patients who received bDMARD within the first 3 months from diagnosis had a significantly greater SMS at presentation compared to those who did not [early-bio: SMS 4 (IQR: 3–5); no early-bio: SMS 2 (IQR: 2–3); *p* = 0.001]. There was no significant difference (*p* = 0.317) in the dose of anakinra received in patients who received this within 3 months of diagnosis (median 2 mg/kg/day; range 1.2–8 mg/kg/day; missing data *n* = 2) vs. those who received it later in their disease course (median 2 mg/kg/day; range 2–6 mg/kg/day; missing data *n* = 2). All patients who received tocilizumab were dosed using standard doses for the intravenous or subcutaneous routes, banded at a 30 kg weight threshold (Supplementary Table S1).

**Table 3:** Comparison of rates of clinically inactive disease in those receiving IL-1 or IL-6 blockade within the first 3-months of diagnosis versus those that did not.

	3-month follow-up *n* = 76		12-month follow-up *n* = 63^e,f^			Last follow-up *n* = 76		
	3.1 months (2.7–3.8)		12 months (11.1–13.3)			4.7 years (2.2–9.2)		
	Early-bio^a^	No early-bio^b^	Early- bio^a^	No early-bio^b^		Early-bio^a^	No early-bio^b^	
Clinically inactive disease^c^	23/40 (58%)	21/36 (58%)	26/29^e^ (90%)	18/34^f^ (53%)		36/40 (90%)	31/36 (86%)	
	ns		*p* = 0.002			ns		
Treatment-free clinically inactive disease (remission)	1/40 (3%)	2/36 (6%)	0/29 (0%)	3/34 (9%)		8/40 (20%)	16/36 (42%)	
	ns		ns			*p* = 0.028		
Glucocorticoid-free clinically inactive disease^d^	7/40 (18%)	7/36 (19%)	20/29 (69%)		16/34 (47%)	34/40 (85%)		30/36 (83%)
	ns		ns			ns		

Median age presented along with interquartile range (IQR). Absolute frequencies presented along with percentages.

^a^ Patients who received either IL-1 or IL-6 blockade within 3 months from diagnosis.

^b^ Patients who did not receive IL-1 or IL-6 blockade within 3 months from diagnosis (irrespective of whether they received those treatments after this time point).

^c^ CID irrespective of current treatment status.

^d^ CID, off glucocorticoids (irrespective of other treatments received).

^e^ Missing data, *n* = 1; time point not reached *n* = 9.

^f^ Missing data, *n* = 2; time point not reached *n* = 1. ns (not significant) at *p* < 0.05 using Fisher's exact test. Shaded cells denote significantly different comparisons.

At 1-year follow-up, CID was achieved in a significantly greater proportion of patients treated with a bDMARD within 3 months of diagnosis compared to those who were not (early-bio CID 90% vs. no early-bio 53%, *p* = 0.002). There was also a higher rate of glucocorticoid-free CID in the early-bio group (69% vs. 47%) at 12 months follow-up, although this did not reach statistical significance (*p* = 0.125).

At the last follow-up (median 4.7 years), high rates of CID were achieved irrespective of timing of biologic initiation (early-bio 90%; no early-bio 86%; *p* = 0.728), and with similar glucocorticoid-free CID rates between the two groups. In contrast, at last follow-up, treatment-free CID (remission rate) was significantly greater in the “no early-bio” group (early-bio CID 20% vs. no early-bio CID 42%, *p* = 0.028).

## Adverse events and mortality

### Growth at last review

Median height centile for the whole cohort at last review was 59.4% (IQR: 27.2%–84.8%); median height *z* score was 0.24 (IQR: −0.6 to 1.0). Median BMI for the whole cohort at last review was 19.2, IQR: 16.5–22.8 (centile 69.1%, IQR: 40.1%–93.5%); median BMI *z* score was 0.51 (IQR: −0.23 to 1.6).

For the bio-early group, at last review, median height *z* score was 0.25 (IQR: −0.6 to 1.2), *n* = 36; for the bio-late group, median height *z* score was 0.24 (IQR: −0.5 to 0.79), *n* = 37 (missing data *n* = 3). Thus, there was no difference in final height acquisition between the bio-early and bio-late group, *p* = 0.71138.

Median BMI for the bio-early group was 18.5, IQR: 16.0–20.9 (median centile 65.9%, IQR: 31.3–94.8); median BMI *z* score was 0.4 (IQR: −0.5 to 1.6). For the bio-late group, median BMI was 20.6, IQR: 17.6–23.1 (median centile 71.2%, IQR: 45.4–93.1); median BMI *z* score was 0.6 (IQR: −1.3 to 3.0). Thus, there was no statistical difference in final BMI between the bio-early and bio-late group, *p* = 0.48392.

### Macrophage activation syndrome

MAS (confirmed or suspected) was documented at first presentation in 36% (*n* = 27/76) of patients. A total of 35/76 (46%) patients had at least one episode of MAS throughout their disease course; two of these patients had two episodes and one patient had three. Of these 35 patients, MAS was diagnosed based on fulfilling the 2016 MAS classification criteria (27) in 23/76 (30%). The remaining 12 patients (16%) had suspected MAS based on documentation by the medical team in the electronic records; 10 of the suspected MAS cases had insufficient documentation to fully check the MAS criteria; the remaining two patients had fever, hyper-ferritinaemia, but only one other criterion; AST >48 U/L and triglycerides >156 mg/dl, respectively.

Patients presenting with MAS were significantly (*p* = 0.026) more likely to receive bDMARD at diagnosis (*n* = 15/27, 56%; anti-IL-1 *n* = 14, anti-IL-6 *n* = 1) compared to those that developed MAS later in their disease course (*n* = 1/8, 1%; anti-IL-1 *n* = 1, anti-IL-6 *n* = 0). All patients presenting with MAS received treatment with glucocorticoids (intravenous, *n* = 26; oral, *n* = 1). Additional treatments for those presenting with MAS included etoposide (*n* = 1, 4%) and cyclosporine (*n* = 9, 33%); two children received a monoclonal antibody against INF-γ (emapalumab) as part of a clinical trial (30, 31).

In relation to disease outcome (CID irrespective of treatment at 3 months, 12 months, and last review), there was no difference between those with or without macrophage activation syndrome at presentation (Supplementary Tables S2–S4).

### Lung disease

One patient (1%) had an abnormal high resolution CT (HRCT) thorax at diagnosis. This 14.1-year-old female presented with MAS with patchy ground glass changes of the lungs bi-basally. She was treated with bDMARD (anti-IL1, anakinra) within 3 months of diagnosis, and at last review, 2.7 years since diagnosis, was in remission (CID off all treatment).

Four months after data collection was completed, we observed a case of pulmonary alveolar proteinosis (PAP) in a 17-month-old girl with sJIA and smouldering MAS (Supplementary Figure S2). She presented with treatment refractory sJIA complicated by MAS at the age of 8 months. She remained glucocorticoid dependent throughout her disease course and received anti-IL6 and anti-IL1 blockade, in addition to cyclosporine, before progressing to etoposide as a bridge to allogeneic-HSCT.

Neither patient had any documented idiosyncratic reaction following bDMARD; and HLA testing was not performed in any of the patients.

### Haematopoietic stem cell transplantation

Four patients underwent HSCT: three allogeneic-HSCT and one autologous-HSCT. Median age at diagnosis of sJIA was 1.8 years (range 0.9–12.8 years, 50% female). Three of the four patients received methotrexate as first-line DMARD.

Regarding the three patients requiring allogeneic-HSCT, the oldest child (female), aged 12.8 years at diagnosis, presented with MAS and was commenced on intravenous glucocorticoids, anakinra and cyclosporine. She required etoposide as a bridge to allogeneic-HSCT within the first year of diagnosis. The remaining two children that received allogeneic-HSCTs were 0.9 years (female) and 1.1 years (male) at diagnosis with sJIA. Both developed MAS and proceeded to allogeneic-HSCT after 2 years (failed sDMARD, anti-TNF, and IL-1 blockade) and after 5 years (failed sDMARD, IL-1 blockade, IL-6 blockade, and required etoposide as a bridge to HSCT), respectively, post their sJIA diagnosis.

Autologous-HSCT was performed 3 years after diagnosis in a male patient, diagnosed with sJIA at the age of 18 months. He had failed treatment with sDMARD and bDMARD including IL-1 blockade and anti-TNF. His sJIA relapsed 2 years after HSCT. He commenced IL-6 blockade. Nine years later, he developed MAS following a presumed viral trigger and was commenced on anakinra.

At last review, all four post-transplant patients had CID; two were in remission off all treatments. One child required the addition of methotrexate for hip arthritis and the other IL-1 blockade (anakinra) due to an episode of MAS post autologous-HSCT.

### Mortality

No patients died during the data collection period, although one patient subsequently died of MAS 7 months after data collection was over. Demographics and specific features of that case are redacted to maintain anonymity.

## Discussion

We describe a large, single-centre cohort of patients with sJIA. Although the spectrum of disease in our patient population was rather severe as indicated by 36% having MAS at first presentation and with high systemic manifestation scores, we found that overall sJIA prognosis was good with 88% of patients achieving CID at last follow-up of median 4.7 years, the majority of whom were glucocorticoid-free (84%). Furthermore, we observed an overall remission rate of 32% (CID, off all treatment) at last follow-up. Outcomes at 1 year were more favourable in those receiving early biologic treatment (within 3 months of diagnosis) compared to those who did not, CID being achieved in 90% vs. 53% (*p* = 0.002), respectively, despite those receiving early biologic treatment having more severe systemic disease. This favourable therapeutic signal was not apparent at last follow-up, however, possibly because most patients (84%) had received bDMARDs by that stage. Other prognostically encouraging observations were: (1) a low percentage of patients requiring HSCT (5%); (2) low rate of sJIA-LD (1%); and (3) no deaths during the data collection period. The one death we did observe occurred in the context of MAS after the data collection period was over but serves to emphasise the potentially serious nature of that complication despite the use of modern therapeutics. Finally, growth was well-preserved in our cohort, with median height *z* score of 0.24 at last review.

Unchecked early autoinflammation in sJIA driven by increased innate immune activation, including dysregulation of IL-1 and IL-6, may lead to a state of chronic T-lymphocyte activation with an unfavourable balance of T-effector and regulatory cells that can drive chronic arthritis over time (4). Early cytokine antagonist therapy could abrogate the development of a population of these arthritis-causing T cells to avert chronic systemic inflammation and arthritis (6, 32, 33). Our observation of higher CID at 1 year in those receiving bDMARD within 3 months of sJIA diagnosis could be compatible with a therapeutic “window of opportunity” hypothesis (34). However, if that were the case, we might have expected higher rates of CID to have persisted in those receiving early biologics over longer follow-up, which in fact was not observed in our cohort. Accordingly, it is difficult to over-interpret our observations since most patients (84%) did eventually receive bDMARDs (mainly anakinra or tocilizumab), which probably diluted any longer-term therapeutic advantage conferred by the early use of bDMARDs.

Liberal use of bDMARDs over the disease course in this cohort may also explain the overall low degree of chronic arthritis (5%) observed at last review. It is also worth noting that due to the natural history of sJIA, a minority of patients with sJIA will remit spontaneously (32), further confounding the interpretation of this retrospective review. In that context, we also noted significantly more treatment-free remission at the last follow-up in those who did not receive bDMARDs in the first 3 months of treatment (42% vs. 20%, *p* = 0.028). This is probably explained by those patients having milder disease as indicated by the lower SMS in this group [early-bio: SMS 4 (IQR: 3–5); no early-bio: SMS 2 (IQR: 2–3); *p* = 0.001]. We emphasise that outside clinical trials, there are multifactorial issues that can contribute to considerable heterogeneity around the timing of treatment withdrawal. Inferring firm conclusions about disease course based on treatment status should thus be considered with some caution for retrospective studies of this nature.

What our observations and the evidence base do strongly suggest, however, is that, it is logical to use proven, effective, and safe treatments for sJIA, i.e., IL-1 or IL-6 blockade, sooner rather than later, and particularly early in the disease course when systemic features dominate the clinical picture. Since 84% of our patients ultimately required this, current NHS pathways in England (15, 35) that mandate failing one sDMARD for 3 months in the case of sJIA, or two sDMARDs in the case of AOSD, are increasingly outdated and not supported by clinical trial data (18), resulting in significant delay in achieving CID. This approach (ironically) is likely to be even more expensive for society when the full economic costs of failing to control a severe systemic inflammatory condition over several months are considered.

Historically, it was reported that about 10% of children with sJIA develop MAS; however, it is more recently suggested that many more patients with sJIA (30%–40%) have subclinical features of MAS (36, 37). Applying the 2016 MAS classification criteria retrospectively to the patients on whom we had available data to do so, we found that 23/76 fully met the criteria, i.e., 30% of the overall cohort at some stage over their disease course. This suggests that our cohort is comparable to the expected rate of MAS more recently appreciated, highlighting the need for ever-vigilant awareness of the risk of MAS in sJIA. As one might expect, and according to the NHS England clinical commissioning policy (15), children presenting in MAS were significantly more likely to receive bDMARD early compared to those who developed MAS later in their disease course.

A full discourse on the pathogenesis and management of MAS is beyond the scope of our cohort review; however, we highlight overall successful outcomes with the early use of anakinra combined with high-dose glucocorticoids. Two patients in our cohort were recruited into an open-label, single-arm emapalumab pilot study, a fully human anti-IFN-γ monoclonal antibody. Favourable outcomes for this treatment are now reported in full elsewhere (31). At the time of writing, a second worldwide trial of this treatment for MAS is ongoing which may in the future provide sufficient evidence to achieve licencing of emapalumab for MAS, as is the case for primary HLH (38).

Three of our four patients who required HSCT received allogeneic-HSCT, an approach now favoured over autologous-HSCT for refractory sJIA (39). The goal of allogeneic-HSCT is to achieve life-long disease remission by replacement of the patients’ dysregulated immune system (40). Unfortunately, relapses post procedure are well described so this is not curative for all (41). Of the four children who received HSCT, two (50%) relapsed post-HSCT (*n* = 1, allogeneic; *n* = 1, autologous) requiring re-instigation of immunosuppressive therapy, on which they achieved CID at last review. This emphasises that HSCT may moderate the disease phenotype to make it more manageable in some cases. Although there could be a role for combining bDMARDs to achieve disease control in refractory cases, we currently have no experience of such an approach and worry that such a strategy may result in infectious complications and contribute to co-morbidities that could jeopardise the success of allogeneic-HSCT that may still ultimately be required (42).

Although over the time course of this retrospective review we did not routinely screen for lung disease, one patient (1%) in our cohort developed sJIA-LD. She presented with MAS and received bDMARD within 3 months of diagnosis. The prevalence of sJIA-LD has been reported to be 6%–8%, with a mortality rate of 37%–68% (28). Our current practice does not dictate that all patients with sJIA are screened for sJIA-LD, due to concerns of radiation exposure associated with CT thorax. Therefore, we may have under-reported sJIA-LD in our cohort. sJIA-LD is characterised by the presence of chronic life-threatening pulmonary pathology including pulmonary hypertension, interstitial lung disease, pulmonary alveolar proteinosis, and/or endogenous lipoid pneumonia (28). Kimura et al. noted that the emergence of chronic lung disease in sJIA coincided with the introduction of IL-1 and IL-6 blockade and decreasing use of glucocorticoids, suggesting the potential contribution of drug exposure (43). Other observed risk factors include age at sJIA onset <2 years, recurrent MAS, prominently elevated IL-18 levels, and IFN-γ-mediated alveolar macrophage activation (44). These observations strongly suggest overlapping pathophysiological pathways between MAS and sJIA-LD. Indeed, Schulert et al. found a strong IFN-γ-induced gene signature in lung biopsy tissue of patients with sJIA-LD (44). With this understanding, it may be easy to see why early case reports support the use of Janus Kinase inhibitors (JAKi) in sJIA, MAS, and sJIA-LD (45).

Our results are limited by all the caveats around retrospective case notes review. It has been reported that insufficiency fractures occurred in 8% of patients with sJIA at Boston Children's Hospital (46). We were unable to reliably report on fracture rates (let alone the mechanism of injury to accurately classify this as insufficiency fracture or not) in our cohort since we were concerned that this was incompletely documented in the medical records. We are thus unable to assess the potential impact of early biologic use on insufficiency fracture in our series.

However, our cohort represents the largest UK series of sJIA described to date, and our summative real-world therapeutic experience is in line with data from clinical trials and other clinical studies of early use of bDMARDs in sJIA (6, 8, 10–13, 46). Based on our experience and the mounting evidence base, we conclude that the early use of bDMARDs is logical and advantageous because it is highly efficacious, safe, and ultimately likely to be cost-saving for society by achieving CID earlier. There is increasing evidence that the efficacy for methotrexate for the systemic features of sJIA is poor. This is reflected in current practice and the recently updated American College of Rheumatology (ACR) guidelines, which recommend first-line IL-1 or IL-6 blockade even in the absence of MAS (47). Evidence for the benefit of IL-1 or IL-6 blockade in sJIA is mounting; De Benedetti et al. reported an 85% ACR30 response rate at week 12 with tocilizumab (IL-6 blockade) compared to a 24% placebo response rate in sJIA (10). The ANAJIS trial, a small randomised, placebo-controlled trial of anakinra (IL-1 blockade) in patients with sJIA, reported 67% ACR30 response at 1 month compared to 8% of the control group (11). Ter Haar et al. report a 55% remission rate for patients with sJIA at 1 month treated with anakinra monotherapy without glucocorticoids, and a 76% remission rate at 1 year, with 52% off treatment (48). Other uncontrolled studies have also demonstrated that the use of anakinra early in the disease course is associated with a very good short-term outcome (26, 49, 50). These are certainly encouraging observational data, although an important caveat is that 1-month assessment of disease activity may not be helpful because of potential for inclusion of patients with monophasic disease course who may remit spontaneously by then irrespective of treatment. There is also increasing evidence for the anti-interleukin-1β antagonist, canakinumab. Two phase 3 trials reported by Ruperto et al. showed ACR30 response of 84% compared to 10% placebo in patients with sJIA (12). Direct comparison of bDMARDs and sDMARDs in patients with AOSD showed beneficial effect of anakinra over sDMARDs in the open-label extension phase (19).

Newer treatments for sJIA that may soon emerge include Janus kinase inhibitors (JAKi). Treatment of sJIA with JAKi is currently being studied in several different clinical trials and reported anecdotally by some to be efficacious and safe (51).

**Figure 1:**
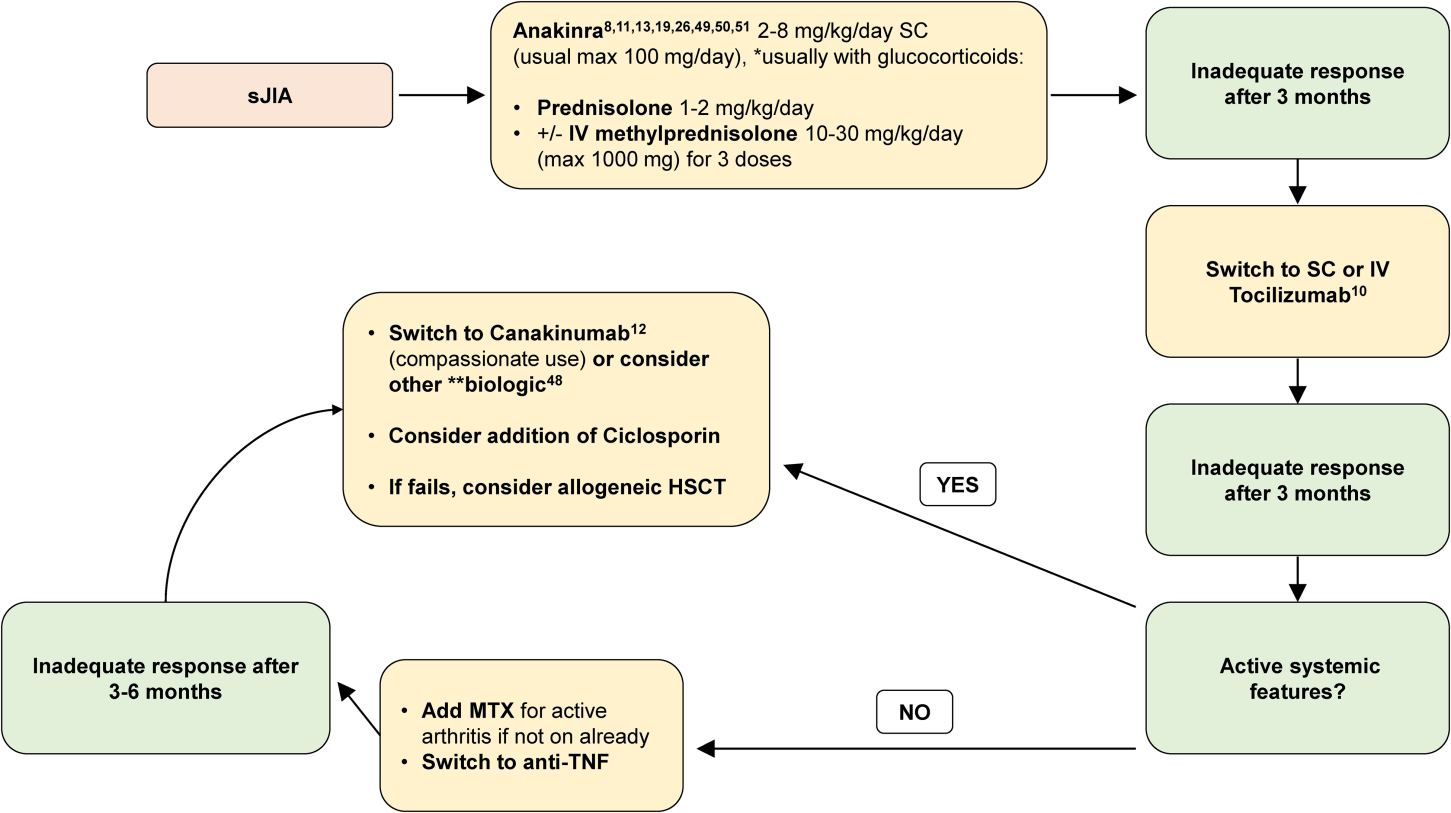
Recommended treatment algorithm for sJIA. max, maximum; IV, intravenous; SC, subcutaneous; MTX, methotrexate; anti-TNF, anti-tumour necrosis factor. Methotrexate (MTX; oral or subcutaneous) may be used at any stage in sJIA particularly if there is evidence of polyarticular arthritis. *Glucocorticoids are usually administered with anakinra for newly presenting patients; however, anakinra may be considered as monotherapy for select patients under close monitoring and expert review (48). **Alternative biologics to consider: anti-TNF, abatacept, or other. Reports of small molecules JAKi have also suggested efficacy and safety (51), but ongoing clinical trials of this treatment for sJIA have not yet completed; therefore, JAKi cannot be recommended with a high level of evidence.

In conclusion, in line with the 2021 ACR guideline for treatment of sJIA (47), the soon-to-be-published European guidance (52), and based on the growing evidence base, we now propose a treatment algorithm for children diagnosed with sJIA in the UK (Figure 1), and strongly suggest that the current NHS England commissioning policy requires urgent reappraisal.

## Data Availability

The raw data supporting the conclusions of this article will be made available by the authors, without undue reservation.
