# Supplementary material for: Systemic juvenile idiopathic arthritis: The Great Ormond Street Hospital experience (2005–2021)
Source: Front Pediatr. 2023 Sep 12;11:1218312. doi: 10.3389/fped.2023.1218312 (PMC10536248; doi:10.3389/fped.2023.1218312)
Supplement: Supplementary file 1 [file Datasheet1.pdf]

Supplementary Figure 1: Systemic Manifestation Score (SMS)

Clinical Manifestation	Points
Fever<br>• 37 – 38 ^oC<br>• 38 – 39 ^oC<br>• 39 – 40 ^oC<br>• > 40 ^oC	 1<br>2<br>3<br>4
Evanescent erythematous rash	1
Generalised lymphadenopathy	1
Hepatomegaly and / or splenomegaly	1
Serositis	1
Anaemia (Haemoglobin < 9 g/dL)	1
Platelet count > 600 x 10^9/L or Ferritin > 500 ng/mL	1

Supplementary Figure 2

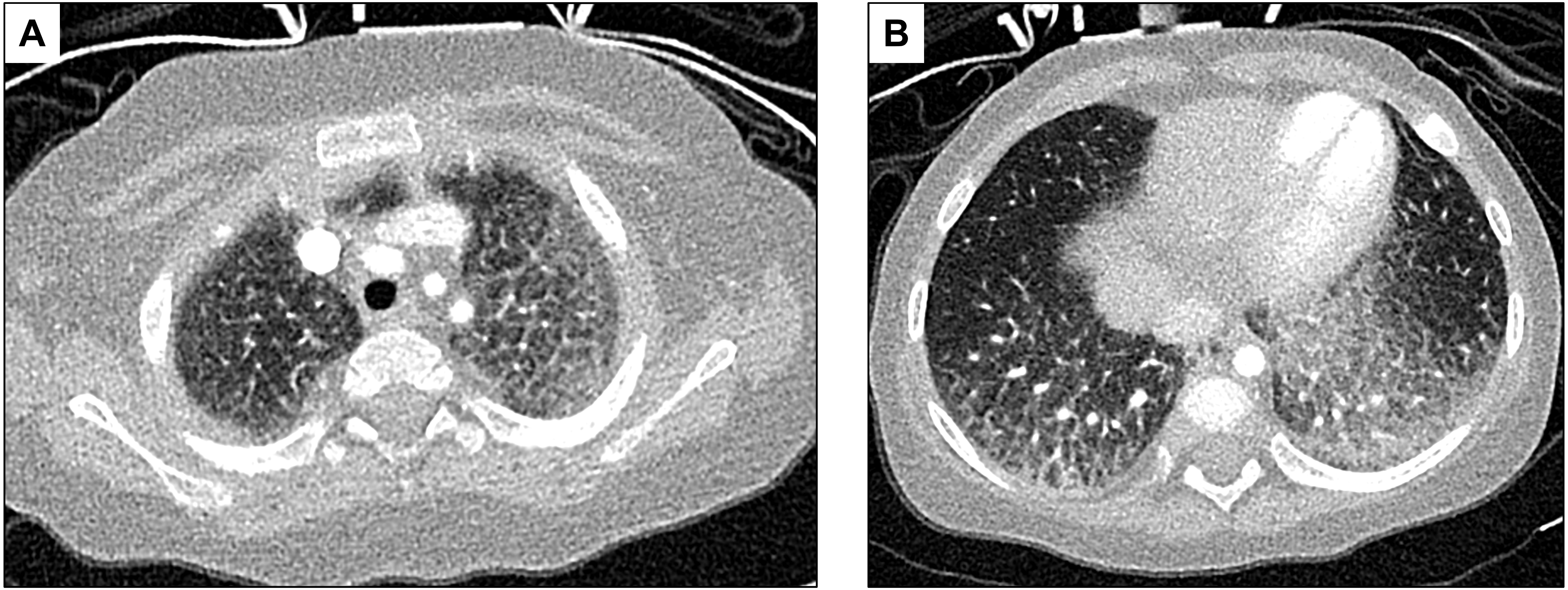

High resolution CT (HRCT) thorax with contrast depicting pulmonary alveolar proteinosis in a 17-month-old child with systemic juvenile idiopathic arthritis and chronic (smouldering) macrophage activation syndrome. (A) Diffuse widespread ground glass opacities with superimposed smooth interlobular septal thickening giving a typical "crazy paving pattern" appearance, characteristically seen in pulmonary alveolar proteinosis. (B) Left lower lobe consolidation with smooth interlobular septal thickening in the bases.
