# Supplementary material for: Systemic juvenile idiopathic arthritis: The Great Ormond Street Hospital experience (2005–2021)
Source: Front Pediatr. 2023 Sep 12;11:1218312. doi: 10.3389/fped.2023.1218312 (PMC10536248; doi:10.3389/fped.2023.1218312)
Supplement: Supplementary file 2 [file Table1.pdf]

Supplementary table 1: Tocilizumab dosing regimen used for all patients receiving Tocilizumab

	Patient weight	sJIA / MAS	pJIA	TA / Vasculitis
Intravenous[1] route	< 30kg	12mg/kg every 2 weeks	10mg/kg every 4 weeks	12mg/kg every 2-4 weeks
	≥ 30kg	8mg/kg (max. 800mg) every 2 weeks	8mg/kg (max. 800mg) every 4 weeks	8mg/kg (max. 800mg) every 2-4 weeks
Subcutaneous[2] route	< 30kg	162mg every 2 weeks (patients ≥ 1 year and ≥ 10kg)	162mg every 3 weeks (patients ≥ 2 years and ≥ 10kg)	162mg every 2-3 weeks (patients ≥ 2 years and ≥ 10kg)
	≥ 30kg	162mg weekly	162mg every 2 weeks	162mg every 1-2 weeks

1. Summary of Product Characteristics –RoActemra 20mg/ml Concentrated Solution for Infusion. Roche Products Ltd. Accessed via https://www.medicines.org.uk/emc
2. Summary of Product Characteristics –RoActemra 162mg Solution for Injection Pre-filled syringe. Roche Products Ltd. Accessed via https://www.medicines.org.uk/emc/

sJIA, systemic juvenile idiopathic arthritis; MAS, Macrophage Activation Syndrome; pJIA, polyarticular juvenile idiopathic arthritis; TA, Takayasu Arteritis

Supplementary table 2: Bivariate analysis of outcome (clinically inactive disease at 3 months) in those with or without macrophage activation syndrome at presentation.

Fisher's exact test p= 0.6288

	**CID at 3 months**	**No CID at 3 months**	**Total**
**Suspected MAS at presentation**	17	10	**27**
**No suspected MAS at presentation**	27	22	**49**
**Total**	**44**	**32**	**76**

Supplementary table 3: Bivariate analysis of outcome (clinically inactive disease at 12 months) in those with or without macrophage activation syndrome at presentation.

Fisher's exact test p= 0.1455

	**CID at 12 months**	**No CID at 12 months**	**Total**
**Suspected MAS at presentation**	19	8	**27**
**No suspected MAS at presentation**	25	24	**49**
**Total**	**44**	**32**	**76**

Supplementary table 4: Bivariate analysis of outcome (clinically inactive disease at last review) in those with or without macrophage activation syndrome at presentation.

Fisher's exact test p= 1.0

	**CID at last review**	**No CID at last review**	**Total**
**Suspected MAS at presentation**	24	3	**27**
**No suspected MAS at presentation**	43	6	**49**
**Total**	**67**	**9**	**76**
